# Mucoepidermoid carcinoma of parotid gland and membranous nephropathy – differentiation between sclerosing mucoepidermoid carcinoma with eosinophilia and Kimura’s disease

**DOI:** 10.1186/s12882-020-02030-1

**Published:** 2020-08-26

**Authors:** Hayato Fujioka, Tsutomu Koike, Teruhiko Imamura, Kota Kakeshita, Hidenori Yamazaki, Hideharu Abe, Takahiko Nakajima, Koichiro Kinugawa

**Affiliations:** 1 The Second Department of Internal Medicine, University of Toyama, 2630 Sugitani, Toyama, Toyama 930-0194 Japan; 2 Department of Otorhinolaryngology, Head & Neck Surgery, University of Toyama, Toyama, Japan; 3 Department of Diagnostic Pathology, University of Toyama, Toyama, Japan

**Keywords:** Membranous nephropathy, Nephrotic syndrome, Sclerosing mucoepidermoid carcinoma with eosinophilia

## Abstract

**Background:**

When we encounter patients who present with both a neck mass and nephrotic syndrome, both malignancy and Kimura’s disease need to be evaluated as the therapeutic strategies differ vastly between them.

**Case presentation:**

We present the case of a 27-year-old male patient with neck mass and nephrotic syndrome. The presence of both eosinophilia and elevated immunoglobulin E levels were concerning for Kimura’s disease, which is an allergic syndrome defined by eosinophilic granulomas of neck soft tissue along with peripheral eosinophilia. The eventual final diagnosis, however, was sclerosing mucoepidermoid carcinoma of parotid gland with both eosinophilia and membranous nephropathy. Following the surgical resection of the mass, the nephrotic syndrome completely resolved.

**Conclusion:**

Detailed histopathological assessments of both the parotid gland and renal tissue were key aspects of the diagnosis and management to exclude Kimura’s disease.

## Background

Membranous nephropathy can occur in the setting of malignant tumors, and often recovers following definitive treatment of malignancy [1]. Sclerosing mucoepidermoid carcinoma with eosinophilia is a rare variant of mucoepidermoid carcinoma, for which surgical resection is generally recommended as a primary treatment. There are currently no published reports of nephrotic syndrome associated with mucoepidermoid carcinoma. Kimura’s disease, which is a benign syndrome accompanied by eosinophilic granulomas of neck soft tissue often found in young men of east-Asian descent, sometimes accompanies renal disease and can be treated by steroid therapy [2, 3].

We present here a young male patient who suffered mucoepidermoid carcinoma of right parotid grand with localized spread to lymph nodes and secondary membranous nephropathy, both of which had significant eosinophilic infiltration. The presence of peripheral eosinophilia and elevated immunoglobulin E level has a broad differential diagnosis, with vastly different treatment pathways.

## Case presentation

A 27-year-old Japanese male patient without a history of any allergic syndromes was admitted to our institute with bilateral peripheral edema, proteinuria, and swelling of the right parotid gland. Cytology of the parotid gland and lymph node biopsy showed no malignancy, though eosinophilic infiltration in the lymph node was observed. He was diagnosed with nephrotic syndrome with 11.9 g/g of creatinine of proteinuria, 1.2 g/dL of serum albumin, and 420 mg/dL of low-density lipoprotein. Mild peripheral eosinophilia (790 /μL) and elevated immunoglobulin E (6896 IU/mL) were also present. Immunoglobulin G was 294 mg/dL, soluble interleukin 2 receptor was 457 U/mL, C3 was 102.9 mg/dL, C4 was 43.1 mg/dL, and total complement activity was 39 U/mL.

**Fig. 1:**
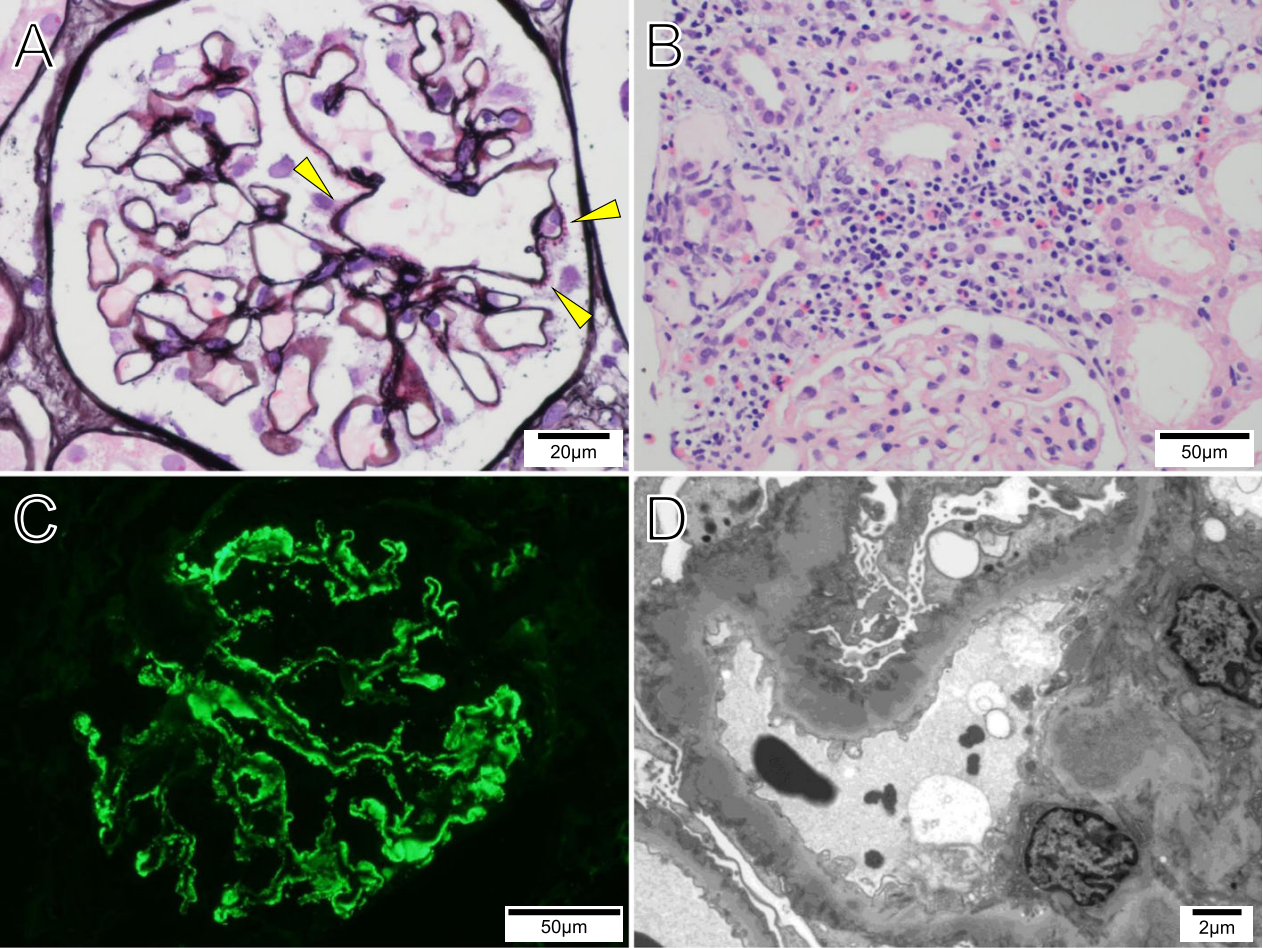
Histopathological findings in kidney biopsy specimen. **a** Diffuse thickness of the glomerular basement membrane with spike formation (arrowhead) (Periodic acid-methenamin-silver stain). **b** Infiltration of eosinophils in renal interstitium (Hematoxylin-Eosin stain). **c** Diffuse granular deposits of immunoglobulin G along the glomerular capillary walls (immunofluorescence stain for IgG). **d** Global subepithelial electron-dense deposits and spike formation of the glomerular baseline membrane (electron microscopy)

Kidney sizes were 112 mm (right) and 119 mm (left). We performed a kidney biopsy to further investigate the mechanism of the observed nephrotic syndrome. On light microscopy, the glomerular basement membrane exhibited mild diffuse thickening with spike formation (Fig. 1a). Eosinophilic interstitial infiltration was also seen (Fig. 1b). Immunofluorescence staining showed diffuse granular deposits of immunoglobulin G and C3 along the glomerular capillary walls (Fig. 1c). Immunoglobulin G4 was not predominant for immunoglobulin G subclass, and kappa and lambda light chains had equal intensity in the immunofluorescence staining. Immunofluorescence staining for the phospholipase A2 receptor (PLA2R) and the thrombospondin type 1 domain-containing 7A (THSD7A) were negative. The electron microscopy showed global subepithelial electron-dense deposits and spike formation of the glomerular baseline membrane (Fig. 1d). He was diagnosed with stage II secondary membranous nephropathy.

**Fig. 2:**
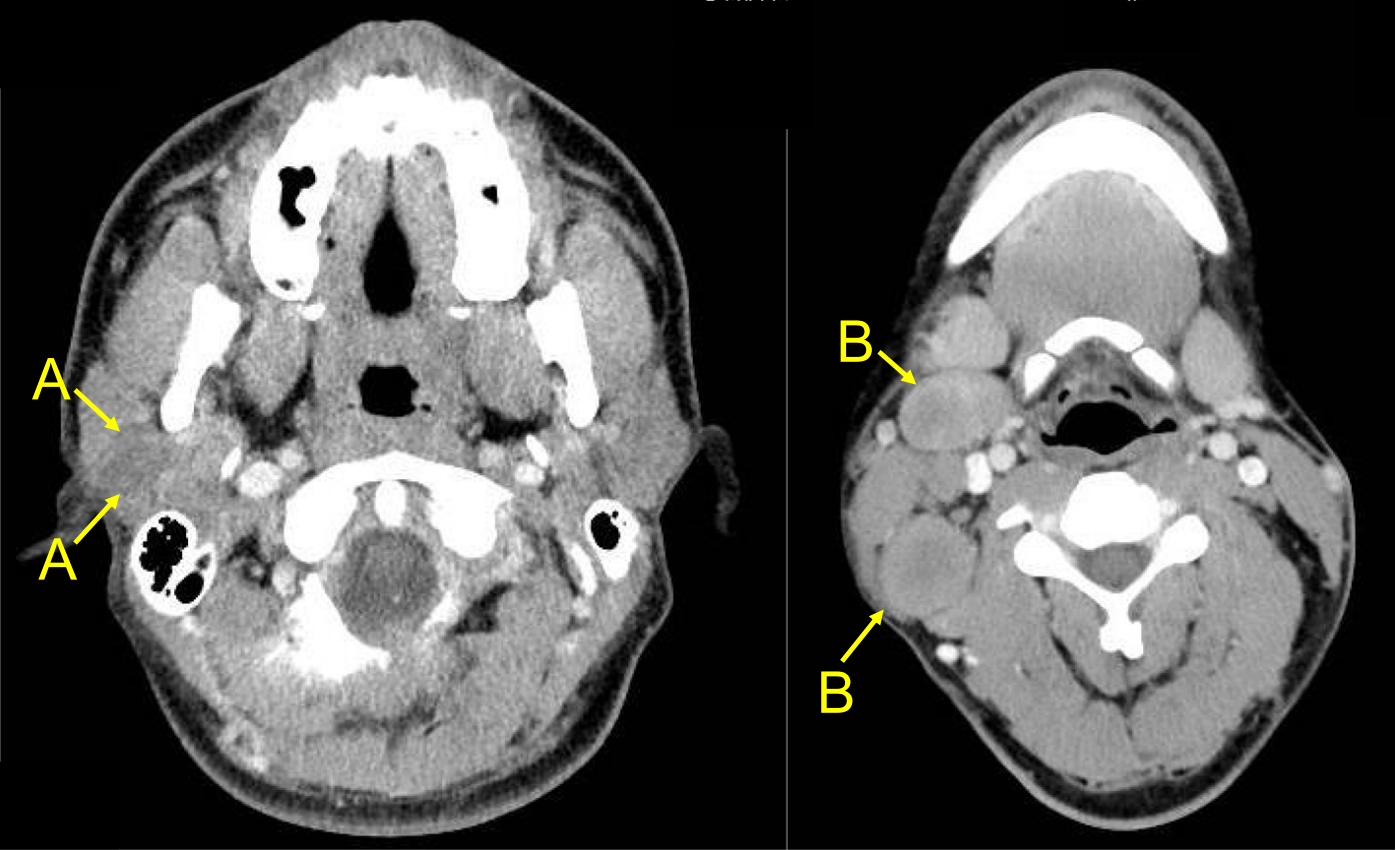
Head computed tomography shows tumor at right parotid gland (**a**) and lymphadenopathy of right neck (**b**)

Computed tomography showed a 40 mm of tumor in the right parotid grand, accompanied by a 23 mm lymphoid focus (Fig. 2), both of which showed uptake by fluorodeoxyglucose-position emission tomography. We at this point suspected malignant disease, instead of an alternative benign presentation such as Kimura’s disease. Repeat biopsies eventually demonstrated carcinoma and lymph node metastasis (T3N2M0, stage IVa).

**Fig. 3:**
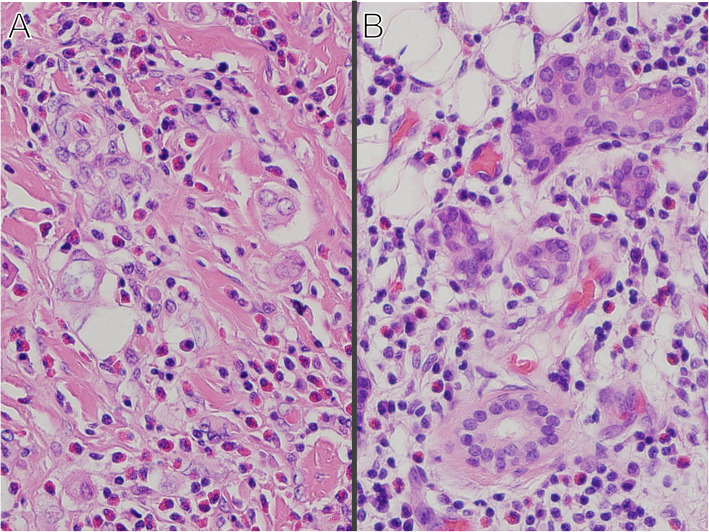
**a** Tumor of right parotid gland (Hematoxylin-Eosin stain) showing invasion of tumor cells, surrounded by sclerotic stroma and numerous eosinophils. **b** Normal tissue of right parotid gland (Hematoxylin-Eosin stain) also showing infiltration of eosinophil

Before surgical excision, steroid pulse therapy (methylprednisolone 500 mg × 3 days) was performed, leading to the partial reduction of proteinuria and eosinophilia. At one month following pulse therapy, the patient underwent successful right parotidectomy with neck dissection. The resected specimen was consistent with the diagnosis of sclerosing mucoepidermoid carcinoma with eosinophilia, a variant of mucoepidermoid carcinoma (pT3N2b) (Fig. 3). The proteinuria achieved complete remission and eosinophilia normalized without steroid therapy during the 12-month follow-up with concomitant post-operative radiation therapy (60 Gy /30fr). He has had no recurrence of disease over two-year follow-up.

## Discussion and conclusion

We diagnosed mucoepidermoid carcinoma with eosinophilia and excluded Kimura’s disease with successive biopsies and detailed histopathological assessment. The patient did clinically well following careful exclusion of Kimura’s disease, for which the correct therapeutic pathway was surgical resection instead of steroid therapy. Both Kimura’s disease and mucoepidermoid carcinoma with eosinophila have several common characteristics, including neck mass with lymph node enlargement, eosinophilia, and high serum immunoglobulin E level.

Mucoepidermoid carcinoma is the most common malignancy among salivary gland-origin tumors, and although rare, it can occur in younger patients [4]. Mucoepidermoid carcinoma has several variants. Sclerosing mucoepidermoid carcinoma with eosinophilia is one of more rarely encountered variants [5]. The exact mechanism of why this carcinoma is associated with peripheral eosinophilia and infiltration is uncertain. Prior studies have suggested that tumor-derived cytokine release might be an important pathobiological mechanism [6–8].

Kimura’s disease is a rare syndrome accompanied by eosinophilic granulomas of neck soft tissue, peripheral eosinophilia, and high immunoglobulin E level. It is most often observed in young East-Asian males [2, 3]. This is a benign chronic granulomatous disease characterized by follicular lymphomas with eosinophilic infiltration. The disease can be treated by steroids or other forms of systemic immunosuppression [9]. Prior reports suggest that renal disease with nephrotic syndrome may occur in up to 16% of patients with Kimura’s disease [10].

The pathogenesis of Kimura’s disease remains unknown. Trauma, infection, an immunoglobulin E-mediated hypersensitivity reaction, or an autoimmune process might stimulate cytokine release that stimulates eosinophil production [11]. Of note, there are no published reports of Kimura’s disease in the presence of a confounding malignancy. In this case, atypical change with eosinophilic interstitial infiltration and negative expression of PLA2R and THSD7A on renal biopsy suggested secondary membranous nephropathy instead of a de novo origin [12].

To our knowledge, this is the first published report of mucoepidermoid carcinoma associated with membranous nephropathy. It is well known that patients with malignancies may have secondary membranous nephropathy (5–22%) [1]. Cases have been reported in lung, prostate and colon cancers, and even with hematologic malignancies [13]. The mechanism of why malignancy-related secondary membranous nephropathies can occur remains unknown, but several hypotheses are proposed [14]: (1) antibody stimulation from tumor antigens immunologically similar to an endogenous podocyte antigen, leading to in situ immune complex formations; (2) shed tumor antigens might form circulating immune complexes that are subsequently trapped in the glomerular capillary wall; (3) circulating antibodies might also react to the tumor antigens that have already been planted in the subepithelial location; and (4) an extrinsic process including viral infection or underlying abnormal immune response might be responsible for both diseases. In this case, a tumor-associated immunoreaction might have had a considerable impact on the development of a secondary membranous nephropathy. The detailed mechanism of renal interstitial infiltration of eosinophils continues to remain uncertain, though tumor-secreted cytokine release might be the causative pathway.

## Data Availability

All the date supporting our findings is contained within the manuscript.

## Abbreviations

PLA2R: Phospholipase A2 receptor
THSD7A: Thrombospondin type 1 domain-containing 7A
